# Difficulties and advances in access to and use of health services by transgender women and travestis in Brazil

**DOI:** 10.1590/1980-549720240007.supl.1

**Published:** 2024-08-19

**Authors:** Thiago Félix Pinheiro, Paula Galdino Cardin de Carvalho, Gabriel Nolasco, Lorruan Alves dos Santos, Maria Amélia de Sousa Mascena Veras

**Affiliations:** I Santa Casa de São Paulo, School of Medical Sciences – São Paulo (SP), Brazil.; II Universidade Católica Dom Bosco – Campo Grande (MS), Brazil.; III Universidade de São Paulo, School of Medicine – São Paulo (SP), Brazil.

**Keywords:** Transwomen, Travestis, Transsexualism, Transphobia, Health services, Barriers to access to health services

## Abstract

**Objective:**

To understand the narratives of transgender women and *travestis* (TGW) from four Brazilian cities regarding access to and use of health services.

**Methods:**

Qualitative study carried out within the scope of the TransOdara project, cross-sectional multicenter mixed methods research conducted between 2019-2021. Fifty-two in-depth interviews with TGW in Manaus, Campo Grande, Porto Alegre and São Paulo were analyzed. The analysis was guided by philosophical hermeneutics.

**Results:**

Reports of discrimination, stigmatization and pathologization reiterate the difficulties faced by TGW in seeking healthcare. The recurrence of disrespect for the social/corrected name reveals obstacles to the recognition of transgender identities and, in some cases, the intention of inhibiting transsexuality-*travestilidade*. Other difficulties arise from actions that disregard the health specificities of TGW or the precarious social conditions that affect some of them. On the other hand, based on experiences of respect and adequate care, participants identify an ongoing change, which is expressed in greater availability of services and improved assistance. There is an expectation of continued expansion of services, technologies and training of health professionals.

**Conclusions:**

The identified change has been undertaken at the interface of public health policies with LGBT+ activism and the production of knowledge about TGW health needs. Although the identified advances are insufficient to change the scenario of the historical exclusion experienced by TGW in health services, they point to promising ways to improve their health conditions.

## INTRODUCTION

In various regions worldwide, transgender individuals^1^ encounter challenges when seeking healthcare. This perpetuates significant social disparities manifested through intersectional discrimination, social marginalization, and violence. Within healthcare settings, they often experience transphobia, stigma, and face a lack of understanding, training, or outright refusal of care from healthcare providers^1^. Past negative encounters or the expectation of such experiences often deter transgender individuals from seeking healthcare services^3^.

In Brazil, despite the limited availability of data on the health status and needs of the transgender population, their heightened vulnerability to various health issues is evident. Specifically, among transgender women and *travestis* (TGW), studies have documented elevated rates of HIV and syphilis infection^4-6^, as well as increased prevalence of anxiety, depression, psychological distress, suicidal ideation, and suicide attempts^7,8^.

Healthcare initiatives targeting TGW were originated from grassroots activism during the HIV/AIDS epidemic, exemplified by organizations like Casa Brenda Lee^9^ in the 1980s. These early initiatives played a crucial role in shaping the government’s response to the epidemic, including funding for Non-Governmental Organizations (NGOs) and tailored interventions for specific populations. However, formal public health policies directed at TGW only began to take shape in the 2000s^10^.

The *National Policy for Comprehensive Health for Lesbians, Gays, Bisexuals, Travestis, and Transsexuals*, developed in 2011, aims to foster equity within the Brazilian Unified Health System (*Sistema Único de Saúde* – SUS) by addressing institutional discrimination related to sexual orientation and gender identity, and its impacts on health, illness, and care processes. Its goals include enhancing access to SUS healthcare services and ensuring access to the process of gender reassignment, initially regulated by the Ministry of Health in 2008 and further defined and expanded in 2013^11^.

The demand for public policies to address the healthcare needs of the transgender population is escalating, given the persistent inadequacies in both public and private health systems to adequately respond to their requirements. There is a clear shortage of services and healthcare professionals equipped to care for this population^12^. Furthermore, access to information about available options remains limited, even regarding services specifically dedicated to gender affirmation^13^.

Transgender individuals in Brazil face significant challenges in accessing and staying in healthcare services, primarily due to the pervasive transphobia present in these settings. Particularly among TGW, there is a prevalent complaint that healthcare professionals often limit their approach to issues related solely to Sexually Transmitted Infections (STIs)^14,15^. This narrow focus serves to reinforce stigmatization. Paradoxically, TGW lack assured access to sufficient prevention and treatment for these infections^13^. For instance, they face multiples barriers in obtaining Pre-Exposure Prophylaxis (PrEP)^16^, despite being prioritized in combination HIV prevention policies. Moreover, stigmatization arises when healthcare professionals unfairly characterize them as promiscuous or presume their involvement in sex work^17^.

Given the advances in visibility and social recognition of the transgender population in Brazil, Monteiro and Brigeiro^18^ observe that discrimination related to gender identity does not necessarily preclude TGW access to services. They employ various strategies to navigate these challenging environments, including adopting intense feminine docility, refining typical middle-class manners, expressing awareness of their rights as citizens, and demonstrating knowledge about the functioning and rules of public institutions.

This study aimed to explore the narratives of TGW from four cities in Brazil concerning their access to and utilization of healthcare services, in light of recent social changes shaping the relationship between the transgender population and the healthcare system. The research investigated memories and reflections on their experiences while seeking healthcare.

## METHODS

This qualitative study is a component of the TransOdara project, a cross-sectional multicenter mixed methods research conducted from 2019 to 2021. The main objective of the project was to estimate the prevalence of syphilis and other STIs among TGW, while also exploring the significance they associate to syphilis and their experiences in accessing and utilizing health services. To this end, the project implemented a point-of-care strategy in collaboration with partner health services, in order to integrate diagnosis, treatment, and prevention of various STIs within the care provided to participants. Additionally, it sought to stimulate, support, and optimize the presence of TGW in these healthcare settings^19^.

This article examines 52 in-depth interviews with TGW who were either undergoing follow-up in the epidemiological component or engaged in other project activities across four cities: Manaus, Campo Grande, Porto Alegre, and São Paulo. It is important to note that the TransOdara project was also conducted in Salvador — data regarding access and utilization of services in that location were previously published by Rossi et al.^14^.

The selection of participants aimed to ensure diversity across categories such as age, education, professional activity (*e.g*., sex workers *versus* other occupations), and current or previous diagnosis of syphilis. The interview guide, in addition to collecting sociodemographic information, addressed topics related to health status, gender transition, experiences with STIs, treatment pathways, and interactions with healthcare professionals and services. Participants were contacted via phone calls and/or messages through applications, utilizing databases from other project activities. Interviews were conducted by trained researchers who were familiarized with the study. In some cases, the interviewer and interviewee were already familiar with each other from previous activities or research endeavors. Interviews in Manaus, Campo Grande, and São Paulo were conducted face-to-face in protected spaces at partner institutions of the project. In Porto Alegre, interviews were conducted over the phone due to restrictions imposed by the worsening of the COVID-19 pandemic. A stipend of R$50.00 was offered to compensate for any expenses related to participation. All interviews were audio recorded, transcribed, reviewed, and subsequently categorized by the authors.

The analytical approach was grounded in epistemological principles derived from philosophical hermeneutics^20^. According to this perspective, the interpretative process involves a dialogue between the discourse generated by the participants, academic literature, and researchers, who serve as interpreters in the recontextualization of meanings. This dialogical process of understanding and interpretation is conceptualized as a “fusion of horizons,” wherein one horizon is merged with another to facilitate mutual accommodation.

The procedure adopted the following steps:

a) comprehensive reading of narratives with the purpose of impregnation, overview and understanding of particularities;
b) categorization of themes;
c) interpretation of the experiences, perceptions, and reflections of the interviewees;
d) articulation with literature; and
e) synthesis of meanings about access to and use of health services.

The project was approved by the Research Ethics Committee of the Santa Casa de Misericórdia de São Paulo (CAAE 05585518.7.0000.5479; opinion n°: 3.126.815 – 30/01/2019), as well as by other participating institutions.The interviewees signed an Informed Consent Form.

## RESULTS

The participants’ ages ranged from 18 to 58 years old, with a mean age of 34 years old. The majority self-identified as *travesti* (21/52) or transgender woman (19/52). Regarding sexual orientation, the majority self-identified as heterosexual (33/52), followed by bisexual/pansexual (6) and homosexual/gay/lesbian (3). In terms of race/color, 25 identified themselves as brown, 12 as black, 10 as white, 1 as indigenous, and 1 as yellow. Concerning to education, 23 had completed secondary education, while the proportions of participants with higher education (15/52) and primary education (14/52) were similar. The majority reported a monthly income ranging from R$1,000.00 to R$2,000.00 (22/52), while 6 had an income of less than R$500.00, and 1 had no income at all. Eighteen participants were engaged in sex work, 4 were students, and 5 were unemployed. Among the remaining participants, the majority worked without a formal contract. Regarding living arrangements, most lived in their own house/apartment (16/52) or rented accommodation (15/52), while 13 lived with their family and 3 lived with friends. Further detailed characterization of each participant is presented in Chart 1.

**Chart 1:** Sociodemographic characterization of the participants.

Site	Fictitious name (age*)	Gender identity	Sexual orientation	Race/color^†^	Education	Monthly income (R$)	Professional occupation
Porto Alegre	Maria (30)	NI	NI	NI	Complete HE	NI	Electrical engineer
	Ana (32)	Trans woman	Heterosexual	Black	Incomplete HS	> 1,000 and ≤ 2,000	Sex worker
	Carolina (58)	Trans woman	Heterosexual	Brown	Incomplete EE	> 1,000 and ≤ 2,000	Retired due to disability
	Jussara (29)	*Travesti*	Homosexual/gay/lesbian	Brown	Complete HE	> 1,000 and ≤ 2,000	Makeup artist/unemployed
	Isabella (40)	Trans (MTF)	Pansexual	Black	Complete HS	> 1,000 and ≤ 2,000	Sex worker
	Laura (49)	*Travesti*	Heterosexual	Black	Incomplete EE	No income	Sex worker
	Heloísa (43)	Trans woman	Heterosexual	Black	Complete HS	> 500 and ≤ 1,000	Retired due to disability
	Fernanda (35)	NI	NI	NI	Complete HE	NI	Religious leader (*Mãe de Santo*)
	Letícia (22)	Trans woman	Bisexual	Brown	Incomplete HS	≤ 500	Unemployed
	Rafaela (27)	*Travesti*	Pansexual	Black	Incomplete HE	> 2,001	Street social educator
	Beatriz (44)	NI	NI	NI	EE	NI	Uber driver
	Marina (31)	Trans woman	Heterosexual	Brown	Incomplete HE	> 1,000 and ≤ 2,000	Housewife
Manaus	Valentina (41)	Trans woman	Heterosexual	White	Complete HE	> 1,000 and ≤ 2,000	Teacher
	Bruna (39)	*Travesti*	Bisexual	Brown	Complete HE	NI	Sex worker
	Amanda (42)	Trans woman	Heterosexual	Indigenous	Complete HS	> 1,000 and ≤ 2,000	Sex worker
	Laís (54)	NI	NI	White	Complete HS	NI	Announcer
	Alice (26)	*Travesti*	Heterosexual	Black	Complete EE	> 1,000 and ≤ 2,000	Sex worker
	Giovanna (19)	NI	NI	Brown	Complete HS	NI	Intern
	Larissa (18)	NI	NI	Brown	Complete HS	NI	Self-employed in the sales area
	Lorena (29)	Trans woman	Heterosexual	Brown	Complete HS	> 500 and ≤ 1,000	Production assistant
	Bianca (42)	*Travesti*	Heterosexual	Brown	Incomplete EE	≤ 500	Hairdresser
	Sabrina (18)	*Travesti*	Heterosexual	Black	Complete HS	≤ 500	Student
	Juliana (45)	NI	NI	Brown	Incomplete EE	NI	Kitchen helper
	Gisele (33)	NI	NI	Brown	Incomplete EE	NI	Sex worker
	Camila (43)	NI	NI	Brown	Incomplete EE	NI	Sex worker
	Daniela (42)	*Travesti*	Heterosexual	White	Incomplete EE	> 500 and ≤ 1,000	Sex worker/cook
	Thais (20)	*Travesti*	Heterosexual	White	Complete HS	> 500 and ≤ 1,000	Student
	Stefani (26)	Trans woman	Heterosexual	Yellow	Incomplete EE	> 1,000 and ≤ 2,000	Student
	Emanuelly (31)	*Travesti*	Heterosexual	Brown	Complete HS	> 1,000 and ≤ 2,000	Sex worker
Campo Grande	Isadora (26)	Female	Other	Black	Incomplete HE	> 2,001	Parliamentary advisor
	Anita (25)	*Travesti*	Homosexual/gay/lesbian	Brown	Complete HS	> 1,000 and ≤ 2,000	Autonomous
	Isabelly (34)	Androgyne/AG/NB	Homosexual/gay/lesbian	Brown	Incomplete HE	> 1,000 and ≤ 2,000	NI
	Lohanna (29)	Trans woman	Heterosexual	White	Incomplete HE	> 1,000 and ≤ 2,000	Saleswoman
	Eduarda (29)	Androgyne/AG/NB	Pansexual	White	Incomplete HE	> 2,001	Photographer
	Lara (29)	Trans woman	Heterosexual	White	Complete HS	> 2,001	House cleaner
	Manuela (20)	*Travesti*	Heterosexual	Brown	Complete HS	> 1,000 and ≤ 2,000	Call center agent
	Gabriela (37)	Trans woman	Heterosexual	White	Incomplete HE	> 2,001	Sex worker
	Lia (27)	*Travesti*	Heterosexual	Brown	Complete HS	> 1,000 and ≤ 2,000	Sex worker
	Lívia (41)	Trans woman	Heterosexual	Black	Incomplete HE	> 1,000 and ≤ 2,000	Teacher
	Yasmin (36)	Trans woman	Heterosexual	White	Incomplete HS	> 1,000 and ≤ 2,000	Unemployed
São Paulo	Rafaelly (20)	Trans woman	Heterosexual	Brown	Complete HS	> 1,000 and ≤ 2,000	Restaurant attendant
	Cláudia (40)	*Travesti*	Bisexual	Brown	Complete HS	NI	Sex worker
	Diana (37)	*Travesti*	Heterosexual	Brown	Incomplete HS	> 1,000 and ≤ 2,000	Sex worker
	Eva (39)	*Travesti*	Heterosexual	Black	Complete HS	> 2,001	Socio-educational advisor
	Fátima (37)	Trans woman	Heterosexual	Brown	Incomplete EE	> 1,000 and ≤ 2,000	Urban cleaning agent
	Janaína (29)	*Travesti*	Heterosexual	Brown	Incomplete HE	> 2,001	Student/Sex worker
	Grace (48)	*Travesti*	Heterosexual	Black	Complete HS	NI	Receptionist
	Guiomar (52)	*Travesti*	Heterosexual	Brown	Incomplete EE	> 500 and ≤ 1,000	Sex worker
	Helena (42)	*Travesti*	Heterosexual	Brown	Incomplete HE	> 2,001	Nursing technique
	Iara (50)	Trans woman	Heterosexual	White	Incomplete EE	> 1,000 and ≤ 2,000	Unemployed/Sex worker
	Ingrid (19)	Trans woman	Heterosexual	Brown	Incomplete HS	> 500 and ≤ 1,000	Unemployed
	Júlia (31)	*Travesti*	Heterosexual	Black	Incomplete EE	> 1,000 and ≤ 2,000	Sex worker

* Complete years at the time of the interview; †Self-reported race/skin color. NI: No information; HE: Higher education; HS: High School; EE: Elementary education; MTF: male to female; AG: Ambiguous gender; NB: Non-binary.

The narratives of the TGW interviewed reveal a significant polarization in their experiences of accessing and using health services. On one side, they recount instances of discrimination, stigmatization, and pathologization of their identities and lifestyles, highlighting a historical pattern of institutional transphobia perpetuated through the attitudes and behaviors of health professionals, as well as in the structure of services. Conversely, they also share positive experiences characterized by respect and adequate care. Through these accounts, participants identify an ongoing shift reflected in the increased availability of services and enhancements in TGW healthcare.

### Difficulties in accessing and using health services

Transphobia manifests through a spectrum of social dynamics within healthcare settings, spanning from subtle glances to overtly discriminatory actions or barriers hindering to assistance. Discrimination is acknowledged as a pervasive reality by interviewees across different demographics, including age, race/color, education, and professional activity. They report experiencing peculiar reactions and subtle forms of discrimination from health professionals or other service users. These encounters not only embarrass TGW but also hinder their ongoing use of services and impact their health by compromising the effectiveness of care (Excerpts 1-4, Chart 2).

**Chart 2:** Selected excerpts from the participants’ narratives

	Participant (city)	Excerpt from the narrative
1	Thais (Manaus)	[I’ve felt discriminated against in healthcare settings] many times. It’s constant, I’m used to it. That’s how it’s gonna be for a long time, and there’s no way to change it. (...) There’s always someone who looks at you, rolls their eyes, gives you a dirty look. You’re feeling fine, having a beautiful day, and then someone looks at you like that, and your day is ruined just because of who you are. And it hurts.
2	Rafaela (Porto Alegre)	I had a consultation with a dermatologist, and I also felt a bit strange. He was kind of curt, like “show me this, show me that.” And then I felt a bit embarrassed about my body.
3	Isabella (Porto Alegre)	A lot of people look at transgender folks differently, always. Let’s say you’re at the health clinic, 10 people go ahead of you, and you end up being the last one. I’ve heard this before, which shouldn’t happen; there should be equality for everyone.
4	Guiomar (São Paulo)	For us, *travestis*, it’s very complicated because you never know how you’ll be treated at that clinic. There is a security guard (...), you don’t know how he’ll receive you because you’re transgender, whether he’ll crack a joke, or be polite. You don’t know how you’ll be treated by the lady who will assist you. So you go in with your head high. You’re already sick, but there’s always someone trying to be funny. (...) They already know they’re going to assist a transgender person, so there is already something stating my name, my birth name, and showing my chosen name underneath. [The doctor] called out [my birth name] loudly, and there were some people there, I heard a few giggles. (...) I want to go back, but who says I want to go back to see him? I don’t. And mind you, I’ve been postponing [my return] for a year now.
5	Eva (São Paulo)	I’ve been through a situation where they looked at me and called me by my father’s name. Even though my name was identified. (...) I waited for her to call out other names, then I stood up, politely asked for permission, and said: “Look, the name you’ve been repeating for a while is my father’s name. My name is Eva. You’ve already checked my ID twice and you’ve noticed that I’m a *travesti*. I’ll go back, sit down, and you will pronounce my name.”
6	Carolina (Porto Alegre)	She faced some restrictions within the hospital, where, at the time, Lucy had to involve the Public Prosecutor’s Office, the whole thing, because they left her in a male area even though she was (...) [gender] reassigned, you know?
7	Janaína (São Paulo)	There are some STI clinics around that I know of, where the girls go, and the doctor, for example, doesn’t specify how PrEP is going to work. If she feels any nausea, when she gets really nauseous because of hormone intake, whether she can take Plasil, or any other medication (...). And we know that the level of education among *travestis* and transsexual populations is not high. (...) I understand, I ask questions, but there are girls who are even ashamed to ask. (...) There are many girls I know, especially in the North Zone, who are minors and die as a result of HIV, actually AIDS. So, I think this issue could also be improved by doing something for the underage girls, who are sex workers, who live off that, (...) who are homeless (...) and have no documents. They’re also denied that [treatment].
8	Laís (Manaus)	I’m always very well attended to when I go to the Emergency Care Service in São Raimundo. (...) I go there because it’s a unit where, every time I arrive, I’ve never been discriminated against and never been denied treatment. They always receive me very well.
9	Eva (São Paulo)	Specialized services are much more humanized than other services, than a Basic Health Unit, than an emergency room, or another healthcare facility. Because specialized services are already more accustomed to this specific population, which I think is the population that tests the most. And they are trained to deal with these people.
10	Manuela (Campo Grande)	I think more things like what happened to me should happen, like someone just showing up and saying “there’s a testing happening there, you have to show up on such-and-such day, such-and-such time” and there will even be an incentive [financial assistance], because I don’t think it’s talked about so much within this community. I think there’s a lack of incentive for the girls to want to get tested.
11	Valentina (Manaus)	I liked it here. In fact, when I came here last month to this project (...), I got vaccinated here on the same day. I found it so quick, I went through the doctors, did my exams, my rapid tests here. I found it very positive, interesting, for you to be alongside the transgender outpatient clinic. I liked it, it’s an incentive to bring other people here, other girls who are out there, outside the system, not getting treatment, not having access, for them to know too.
12	Cláudia (São Paulo)	Back in the day, there was more prejudice, we suffered more. Nowadays, it’s not like that, things are much better for us. We’re having more opportunities to live.
13	Júlia (São Paulo)	Professionals are more attentive because nowadays disrespect doesn’t go unnoticed, the manager calls them into the office and says: “look, darling, this isn’t going to fly because it’s not right; (...) you might be religious at church, but here, this is a health center, this is your job; so, get a grip, respect people.” It’s a matter of respect.
14	Laura (Porto Alegre)	You know, I think nowadays, we’re having a lot more opportunities, you know? For example, just recently, a laboratory opened up. (...) They do all kinds of treatments for transgender people, *travestis*, you know? And they’re really attentive. I loved it. I went there. They’re wonderful with us, you know?
15	Heloísa (Porto Alegre)	What’s lacking is (...) attention, agility in exams for us to know if we have HIV, if we have AIDS, if we have syphilis, HPV, gonorrhea, all these diseases. (...) So, what’s missing is a hormonization and a project that can ensure for us, trans and transsexual individuals, to be treated more, to be cared for more, to be called in more quickly.
16	Grace (São Paulo)	Many girls work on the street and are very exposed. Sometimes, you can’t wait 3, 4 months. They’re out there exposing themselves every day, and it happens, you know, to contract something and need faster treatment. Maybe that’s it, the improvement should be that, having more specialized places for transgender women and *travestis*, and also transgender men.
17	Eva (São Paulo)	So, like, it’s about humanizing the care. (...) If my team doesn’t understand what transgender identity is all about, as the director of this place, I would seek out someone who has greater and better knowledge than my team, to come and train my team. [...] It’s about removing that robotic, automatic thing.
18	Rafaelly (São Paulo)	There should be more treatment for transgender people. More alternatives. Having specialized endocrinologists for transgender people in all clinics, which I think is what’s lacking, they really need to understand what a transgender body is and not think that any medication, anything will help us.
19	Lia (Campo Grande)	It would start with training professionals to receive these individuals. It’s because, often, due to social exclusion, these people end up not seeking healthcare services, and I think that by investing in specific units for the health of transgender people... There is a specificity: hormone therapy, psychological support, and so on.
20	Fernanda (Porto Alegre)	If transgender women were the ones providing [the care], for themselves, by themselves, they wouldn’t feel like aliens in the hospital. If the attendant were a transgender person, if the doctor were a transgender man, they would have a bit more freedom and feel like normal people. I think if they spoke the same language... (...) I think if they opened doors for transgender and gender non-conforming individuals to be attendants, nursing assistants, or even doctors, communication would be a bit clearer.
21	Júlia (São Paulo)	Society owes a debt. [Healthcare for the transgender population] is a priority, yes. There’s this historical portrayal that the public health system has killed a lot of people. So, they’ll have to treat us like queens, yes, at the UBS, to try to make amends, they’ll have to address us as ‘ma’am,’ ‘miss,’ or ‘madam,’ if necessary. And they’ll have to provide quality care. Why? Because *travestis* are people just like everyone else, but the only difference is that we have to keep proving that we’re human.

There are consistent reports indicating that health professionals and other service staff persist in using the name registered at birth, even when they are aware of the preferred name chosen by the TGW. Interviewees note that professionals encounter difficulty or resistance in making adjustments to names, pronouns, and gender references in documents, information systems, and communication within the services. In certain instances, the disregard for the social/corrected name and preferred pronouns not only reflects obstacles in recognizing transgender identities but also suggests an intention to inhibit or penalize transsexuality-*travestilidade* (Excerpt 5, Chart 2).

Additional challenges arise from actions that overlook the unique needs of TGW seeking services. TGW expressed feelings of disrespect and lack of support when receiving care tailored for cisgender individuals or based solely on their assigned gender at birth. Indeed, some accounts reveal indicators of abuse of authority and efforts to assert dominance by imposing specific conditions of care. Similarly, care that fails to address the precarious social circumstances impacting the health and care options of certain TGW becomes constrained or even ineffectual (Excerpts 6 and 7, Chart 2).

### Expansion of services and improvement in care

Positive healthcare experiences highlight efforts to alleviate social and health disparities affecting TGW. Interviewees emphasize that crucial elements for quality care include respect for gender identity and the preparation of healthcare professionals to attend to this population. These criteria prove to be more significant than other factors such as geographic location — leading some TGW to travel to distant neighborhoods to access appropriate care (Excerpts 8 and 9, Chart 2). While Emergency Care and Basic Health Units are mentioned, there is notable recognition of the efforts made in specialized services for STIs/HIV/AIDS or gender transition, specifically concerning the mentioned criteria. This recognition was not attributed to particular professional categories.

The reference to the experiences lived in the partner services of the project from which this study derives highlights the importance of strategies employed for the following purposes:

a) expanding the dissemination of services on TGW networks;
b) making it possible to go to services, especially for those for whom the cost of travel may be an impediment to seeking care;
c) improving the user’s engagement with the service, enhancing the range of technologies available and addressing issues such as delay and fragmentation of interventions — typical problems of the health system, which, in the case of populations less linked to services, can significantly affect the continuity of care (Excerpts 10 and 11, Chart 2).

Positive experiences serve as a contrasting element to the challenges that have historically defined the relationship between transgender individuals and healthcare services. Particularly, older interviewees note significant advancements, including the increasing acknowledgment of this population’s right to healthcare, the expansion of research and discourse on health-related topics, the greater availability of services tailored to their needs, and improvements in professional training to care for this population (Excerpts 12-14, Chart 2).

The interviewees express expectations for the expansion of services, extended operating hours, and increased availability of technologies, along with enhanced care for transgender individuals (Excerpts 15 and 16, Chart 2). They also emphasize the importance of training or educating healthcare professionals as a crucial component of ongoing improvements. This training is seen as vital for fostering more respectful attitudes and actions, as well as for enhancing understanding of transsexuality-*travestilidade* and the technical expertise required to address the healthcare needs of this population (Excerpts 17-19, Chart 2).

The envisioned change in access to and utilization of healthcare services ultimately addresses structural issues that have historically led to social and health inequities, reflected, for instance, in the underrepresentation of transgender individuals in the development and implementation of health policies and actions, resulting in healthcare being predominantly provided by cisgender individuals, often from a cisgender perspective (Excerpts 20 and 21, Chart 2).

## DISCUSSION

The analysis of the challenges encountered by the TGW interviewed regarding access to and utilization of healthcare services reaffirms the findings documented in existing literature. It underscores the extent of health inequities stemming from structural transphobia and its consequential impact on the health and well-being of TGW^1,3,13,21-23^. This highlights an ethical and practical contradiction inherent within healthcare environments. While TGW seek relief or prevention of illness, these spaces frequently manifest as hostile environments, repelling them instead of providing the expected support, thereby exacerbating their vulnerability to various health issues.

Many of these challenges are directly linked to sexual and gender discrimination, although TGW also face obstacles commonly experienced by other users of SUS, including extended waiting times, lack of information, and a shortage of doctors^18^. The persistent disregard for their social/corrected names and gender identities not only denies their established rights^2^ but also hinders their access to services and leads to treatment abandonment^13,14,21,24^.

The manifestations of punishment and subjugation, as recounted by the interviewees regarding the attitudes of healthcare professionals, underscore how gender conceptions and disputes in sexual morality contribute to the social dynamics within healthcare settings. Consequently, the presence of TGW in these spaces often leads to potential tensions and conflicts, deterring them from seeking healthcare services. Systemic transphobia is also evident in the inadequate training and competency of healthcare professionals to address the identities, concerns, and specific healthcare needs of transgender individuals^16,23^. This perpetuates a vicious cycle where societal manifestations of transphobia intersect with the absence of public policies aimed at safeguarding the rights of transgender individuals, further compounded by inadequate readiness of healthcare services and professionals to address their needs. While difficulties in accessing and utilizing services may also intersect with other forms of discriminatory social dynamics, such as racism^25^, this aspect was not explicitly addressed in the interviews.

Positive experiences, on the other hand, signify progress in developing healthcare practices that honor the identities and unique needs of TGW, effectively addressing their healthcare requirements. The recognition that access to and utilization of healthcare services are evolving remains relatively underexplored in the literature. This acknowledgment reflects the recent expansion of services tailored to TGW, as well as improvements in their healthcare experiences and outcomes.

The anticipation for ongoing expansions and enhancements underscores the insufficiency of the progress made thus far in changing the enduring pattern of exclusion experienced by TGW within healthcare services. However, this outlook also reinforces promising avenues for improvement. Recognition of the quality of care received, particularly in specialized services focusing on STIs/HIV/AIDS and gender transition, as well as in the partner services of this project, validates the effectiveness of strategies implemented in these settings to combat discrimination and provide tailored healthcare interventions addressing the unique health needs of TGW, thereby alleviating the social inequities they face.

The observed shift is a product of the intersection between public health policies and LGBT+ activism, coupled with increased awareness and visibility of TGW in Brazil through their political advocacy efforts^18^. This evolution is further fueled by the recent surge in academic discourse surrounding the health issues affecting this population. According to Favero^26^, the emergence of “trans studies” in the 2000s expanded beyond the narrow focus on TGW involvement in sex work, sparking discussions on health and clinical care, particularly regarding the depathologization of gender within diagnostic frameworks. The advent of transfeminism^3^ ushered in a new era of political mobilization for this population, extending beyond the traditional NGO sphere focused on HIV/AIDS advocacy. The growing presence of TGW in academic institutions has facilitated a transition from being subjects of research to active researchers, driving demands for epistemological acknowledgment of their worldviews, including their perspectives on health and well-being. Consequently, several interviewees stress the imperative of dismantling systemic barriers that exclude TGW from participating in the formulation and implementation of healthcare policies and initiatives, often dominated by cisnormative perspectives.

Since this study involved the utilization of healthcare services by the participants, it is conceivable that the reporting of encountered challenges might have been alleviated by the participants successfully surmounting certain potential access barriers. Additionally, despite the implementation of dissemination strategies and invitations to participate, it is plausible that some TGW, particularly those situated within more extreme contexts of vulnerability, such as those with limited social networks or residing in areas with inadequate infrastructure (*e.g*., lacking public transportation, internet access, etc.), may not have been included in the study. Comparisons between the results by region were not possible due to specificities in carrying out the research in each site, especially with regard to the adaptations necessary to face the COVID-19 pandemic.

The narratives examined reveal the intersection of two conflicting movements in the interaction between TGW and healthcare services. This contradiction becomes particularly pronounced against the backdrop of Brazil’s current political and social landscape. The resurgence of conservatism in recent years — characterized by the rise of religious fundamentalism within the government and attacks on gender and sexuality studies and discourse — has led to an escalation of transphobia in its various forms and a curtailment of the rights of transgender individuals^27^. Conversely, the post-pandemic return to democracy, marked by the election of TGW to political office and the reconfiguration of public health and human rights policies, creates opportunities for the achievements made or anticipated to be widely and consistently implemented, thereby effecting tangible improvements in the healthcare outcomes of transgender people.

## Footnotes

1 The authors of *Standards of Care*, produced by the World Professional Association for Transgender Health’s (WPATH), in its eighth version (2022), use the term Transgender and Gender Diverse (TGD) to cover the greatest possible global variety of people with gender identities and expressions that differ from the gender assigned to them at birth^1^. In Brazil, the term trans has been used as an encompassing category of these different gender identities and expressions^2^.

2 Ordinance No. 1.820, of August 13, 2009, of the Ministry of Health, establishes as a right, in the health services network, humanized, welcoming care, free from any discrimination, restriction or denial due to gender identity, among other features. It also resolves that there must be a field in every user’s document to register the social name independent of the civil registry, ensuring the use of the preferred name (Available at https://bvsms.saude.gov.br/bvs/saudelegis/gm/2009/prt1820_13_08_2009.html).

3 Transfeminism consists of a line of feminist thought and practice based on the process of political consciousness and resistance of transgender people, as well as black feminism, mainly on the concepts of intersectionality and non-hierarchy of oppression. Re-discusses the morphological subordination of gender to sex, which has served as a justification for the oppression of people whose bodies do not conform to the binary norm man/penis and woman/vagina^28^.
