# Supplementary material for: Difficulties and advances in access to and use of health services by transgender women and travestis in Brazil
Source: Rev Bras Epidemiol. 2024 Aug 19;27(Suppl 1):e240007.supl.1. doi: 10.1590/1980-549720240007.supl.1 (PMC11338531; doi:10.1590/1980-549720240007.supl.1)
Supplement: Translation

# Translation: Dificuldades e avanços no acesso e no uso de serviços de saúde por mulheres trans e travestis no Brasil

**DOI:** 10.1590/1980-549720240007.supl.1.2

**Authors:** Thiago Félix Pinheiro, Paula Galdino Cardin de Carvalho, Gabriel Nolasco, Lorruan Alves dos Santos, Maria Amélia de Sousa Mascena Veras

**Affiliations:** I Santa Casa de São Paulo, Faculdade de Ciências Médicas – São Paulo (SP), Brasil.; II Universidade Católica Dom Bosco – Campo Grande (MS), Brasil.; III Universidade de São Paulo, Faculdade de Medicina – São Paulo (SP), Brasil.

**Keywords:** Mulheres trans, Travestis, Transexualidade, Transfobia, Serviços de saúde, Barreiras ao acesso aos cuidados de saúde

## Abstract

**Objetivo:**

Compreender as narrativas de Mulheres Trans e Travestis (MTT) de quatro cidades brasileiras acerca do acesso e do uso de serviços de saúde.

**Métodos:**

Estudo qualitativo realizado no âmbito do projeto TransOdara, pesquisa transversal multicêntrica de métodos mistos, conduzida entre 2019 e 2021. São analisadas 52 entrevistas em profundidade com MTT em Manaus, Campo Grande, Porto Alegre e São Paulo. O tratamento analítico foi orientado pela hermenêutica filosófica.

**Resultados:**

Relatos de discriminação, estigmatização e patologização reafirmam as dificuldades enfrentadas por MTT na busca por cuidado com a saúde. A recorrência do desrespeito ao nome social/retificado revela obstáculos ao reconhecimento das identidades trans e, em alguns casos, a intenção de inibir a transexualidade-travestilidade. Outras dificuldades decorrem de ações que desconsideram as especificidades de saúde das MTT ou as condições sociais precárias que afetam algumas delas. No entanto, a partir de experiências de respeito e atendimento adequado, as participantes identificam uma mudança em curso, que se expressa em uma maior disponibilidade de serviços e na melhoria da assistência. Há uma expectativa de continuidade da ampliação de serviços, tecnologias e capacitação dos/as profissionais de saúde.

**Conclusão:**

A mudança identificada tem sido empreendida na interface das políticas públicas de saúde com o ativismo LGBT+ e a produção de conhecimento acerca das questões de saúde das MTT. Os avanços empreendidos, ainda que insuficientes para mudar o cenário da histórica exclusão vivida por elas nos serviços de saúde, apontam caminhos promissores na melhoria de suas condições de saúde.

## INTRODUÇÃO

Em diferentes partes do mundo, pessoas trans^1^ enfrentam dificuldades na busca por cuidados em saúde, o que reforça as graves disparidades sociais expressas em formas interseccionais de discriminação, exclusão social e violência. Nos serviços de saúde, elas comumente vivem experiências de transfobia e estigmatização e se deparam com a falta de conhecimento e treinamento ou mesmo com recusas de atendimento por parte de profissionais da saúde^1^. Experiências negativas prévias ou a antecipação de que essas podem ocorrer têm levado pessoas trans a evitarem buscar serviços de saúde^3^.

No Brasil, apesar da escassez de dados sobre as condições e necessidades de saúde da população trans, é possível identificar sua alta vulnerabilidade a diferentes agravos à saúde. Particularmente entre Mulheres Trans e Travestis (MTT), estudos têm registrado as taxas mais elevadas de infecção por HIV e sífilis^4-6^, bem como altos índices de ansiedade, depressão, sofrimento psíquico, ideação e tentativa de suicídio^7,8^.

Ações de cuidado em saúde direcionadas às MTT foram desenvolvidas a partir de iniciativas de ativistas no âmbito da epidemia de HIV/aids, como a Casa Brenda Lee^9^, na década de 1980. Tais iniciativas foram importantes para o desenvolvimento da resposta governamental à epidemia, especialmente por meio do financiamento de Organizações Não Governamentais (ONGs) e de ações para populações específicas. Entretanto, foi apenas na década de 2000 que políticas públicas de saúde direcionadas a MTT começaram a ser formalizadas^10^.

A *Política Nacional de Saúde Integral de Lésbicas, Gays, Bissexuais, Travestis e Transexuais*, formulada em 2011, propõe promover a equidade no Sistema Único de Saúde (SUS) perante a discriminação institucional por orientação sexual e identidade de gênero e as suas implicações no processo de saúde, adoecimento e cuidado. Entre seus objetivos, constam a ampliação do acesso aos serviços de saúde do SUS e a garantia de acesso ao processo transexualizador, regulamentado pelo Ministério da Saúde em 2008 e redefinido e ampliado em 2013^11^.

A demanda por políticas públicas que promovam a devida atenção à saúde da população trans é crescente, já que os sistemas público e privado de saúde ainda não oferecem respostas suficientes a suas necessidades. É notória, nesse sentido, a escassez de serviços e de profissionais preparados para o atendimento a essa população^12^. Adicionalmente, é restrito o acesso a informações acerca das opções disponíveis, mesmo quando se trata de serviços que trabalham especificamente com afirmação de gênero^13^.

As dificuldades no acesso e na permanência de pessoas trans nos serviços de saúde no Brasil derivam sobretudo da transfobia que encontram nesses espaços. Entre as MTT, é comum a queixa de que a abordagem dos/as profissionais de saúde, muitas vezes, se restringe a questões relativas às Infecções Sexualmente Transmissíveis (ISTs)^14,15^, o que reforça a estigmatização. Contraditoriamente, não lhes é garantido o acesso adequado à prevenção e ao tratamento dessas infecções^13^. O acesso das MTT à PrEP, por exemplo, é restrito por várias barreiras^16^, apesar de constarem como população prioritária nas políticas de prevenção combinada ao HIV. A estigmatização também ocorre quando profissionais de saúde as julgam como promíscuas ou presumem que são sempre profissionais do sexo^17^.

Diante dos avanços na visibilidade e no reconhecimento social da população trans no Brasil, Monteiro e Brigeiro^18^ observam que a discriminação relacionada à identidade de gênero não necessariamente impossibilita o acesso de MTT aos serviços. Para contornar as dificuldades encontradas nesses ambientes, elas recorrem a estratégias tais como a performance de uma intensa docilidade feminina e um refinamento dos modos típicos de classes médias ou a expressão de uma consciência de seus direitos como cidadãs e de conhecimento acerca do funcionamento e das regras das instituições públicas.

Com base nas recentes mudanças sociais que delineiam a relação da população trans com o sistema de saúde, este estudo tem o objetivo de compreender as narrativas de MTT de quatro cidades brasileiras a respeito do acesso e do uso de serviços de saúde. Para isso, investigam-se suas memórias e reflexões acerca das experiências na busca por cuidado em saúde.

## MÉTODOS

Trata-se de estudo qualitativo, integrante do projeto TransOdara, pesquisa transversal multicêntrica de métodos mistos realizada entre 2019 e 2021. O projeto objetivou estimar as prevalências de sífilis e outras ISTs entre MTT, compreender os significados atribuídos por elas à sífilis e suas experiências relacionadas ao acesso e ao uso dos serviços de saúde. Para isso, utilizou a estratégia *point of care* junto aos serviços de saúde parceiros, de modo a integrar diagnóstico, tratamento e prevenção de um conjunto de ISTs no atendimento das participantes, além de estimular, apoiar e otimizar sua presença nesses espaços^19^.

Neste artigo, são analisadas 52 entrevistas em profundidade com MTT em seguimento no componente epidemiológico e/ou participantes de outras atividades do projeto em quatro cidades: Manaus, Campo Grande, Porto Alegre e São Paulo. O projeto TransOdara foi realizado também em Salvador – dados referentes ao acesso e uso de serviços nesse sítio, em particular, foram previamente publicados por Rossi et al.^14^.

A seleção das participantes buscou garantir uma diversificação por categorias de idade, escolaridade, atuação profissional (i.e., profissionais do sexo *versus* outras ocupações) e diagnóstico atual ou prévio de sífilis. O roteiro das entrevistas, além da identificação sociodemográfica, abrangia questões relacionadas a condições de saúde, transição de gênero, experiências com ISTs, itinerários terapêuticos e relação com profissionais e serviços de saúde. As participantes foram contatadas por telefonema e/ou mensagens via aplicativos a partir das bases de dados provenientes de outras atividades do projeto. As entrevistas foram conduzidas por pesquisadores/as treinados/as e previamente familiarizados/as com o roteiro. Em alguns casos, entrevistada e entrevistador/a já se conheciam de atividade ou pesquisa anterior. Em Manaus, Campo Grande e São Paulo, as entrevistas foram realizadas presencialmente em espaço privativo nas instituições parceiras do projeto. Em Porto Alegre, foram realizadas via telefonema devido a restrições impostas pelo agravamento da pandemia de COVID-19. Uma ajuda de custo no valor de R$ 50,00 foi oferecida como ressarcimento para eventuais gastos relacionados à participação. As entrevistas foram gravadas em áudio, transcritas, revisadas e, posteriormente, categorizadas pelos autores.

O tratamento analítico foi orientado pelos princípios epistemológicos decorrentes da hermenêutica filosófica^20^. Nessa perspectiva, o processo interpretativo consiste em um diálogo entre as formas de discurso produzidas pelas participantes, a literatura acadêmica e os/as pesquisadores/as, que atuam como intérpretes na rearticulação de sentidos. A experiência dialógica de compreensão-interpretação é encarada como uma “fusão de horizontes”, na qual um horizonte é acomodado a outro.

O procedimento adotou os seguintes passos:

a) leitura compreensiva das narrativas com propósito de impregnação, visão de conjunto e apreensão das particularidades;
b) categorização dos temas;
c) interpretação das experiências, percepções e reflexões das entrevistadas;
d) articulação com a literatura; e
e) síntese de sentidos acerca do acesso e uso dos serviços de saúde.

O projeto foi aprovado pelo Comitê de Ética em Pesquisa (CEP) da Santa Casa de Misericórdia de São Paulo (CAAE 05585518.7.0000.5479 - Nº parecer: 3.126.815 - 30/01/2019), assim como pelas demais instituições participantes. As entrevistadas assinaram Termo de Consentimento Livre e Esclarecido (TCLE).

## RESULTADOS

A idade das participantes variou de 18 a 58 anos, com média de 34 anos. A maior parte se identificou como travesti (21/52) ou mulher trans (19/52). A maioria afirmou ser heterossexual (33/52); 6, bissexual/pansexual; 3, homossexual/gay/lésbica. Em termos de raça/cor, 25 se identificaram como parda, 12 como preta, 10 como branca, 1 como indígena e 1 como amarela. No que tange à escolaridade, 23 tinham Ensino Médio e as proporções de participantes com Ensino Superior (15/52) e com Ensino Fundamental (14/52) foram similares. A maioria possuía renda mensal de R$ 1.000,00 até R$ 2.000,00 (22/52), 6 possuíam renda menor que R$ 500,00 e 1 não possuía renda alguma. 18 participantes eram profissionais do sexo, 4 estudantes e 5 estavam desempregadas; das demais, a maior parte trabalhava como autônoma. A maioria morava em casa/apartamento próprio (16/52) ou alugado (15/52), 13 moravam com a família e 3 com amigos. A caracterização detalhada de cada participante é apresentada no Quadro 1.

**Quadro 1:** Caracterização sociodemográfica das participantes.

Sítio	Nome fictício (idade*)	Identidade de gênero	Orientação sexual	Raça/cor^†^	Escolaridade	Renda mensal (R$)	Atuação profissional
Porto Alegre	Maria (30)	SI	SI	SI	ES completo	SI	Engenheira elétrica
	Ana (32)	Mulher trans	Heterossexual	Preta	EM incompleto	> 1.000 e ≤ 2.000	Profissional do sexo
	Carolina (58)	Mulher trans	Heterossexual	Parda	EF incompleto	> 1.000 e ≤ 2.000	Aposentada por invalidez
	Jussara (29)	Travesti	Homossexual/gay/lésbica	Parda	ES completo	> 1.000 e ≤ 2.000	Maquiadora/desempregada
	Isabella (40)	Trans (MTF)	Pansexual	Preta	EM completo	> 1.000 e ≤ 2.000	Profissional do sexo
	Laura (49)	Travesti	Heterossexual	Preta	EF incompleto	Nenhuma renda	Profissional do sexo
	Heloísa (43)	Mulher trans	Heterossexual	Preta	EM completo	> 500 e ≤ 1.000	Aposentada por invalidez
	Fernanda (35)	SI	SI	SI	ES completo	SI	Mãe de Santo
	Letícia (22)	Mulher trans	Bissexual	Parda	EM incompleto	≤ 500	Desempregada
	Rafaela (27)	Travesti	Pansexual	Preta	ES incompleto	> 2.001	Educadora social de rua
	Beatriz (44)	SI	SI	SI	EF	SI	Motorista de Uber
	Marina (31)	Mulher trans	Heterossexual	Parda	ES incompleto	> 1.000 e ≤ 2.000	Dona de casa
Manaus	Valentina (41)	Mulher trans	Heterossexual	Branca	ES completo	> 1.000 e ≤ 2.000	Professora
	Bruna (39)	Travesti	Bissexual	Parda	ES completo	SI	Profissional do sexo
	Amanda (42)	Mulher trans	Heterossexual	Indígena	EM completo	> 1.000 e ≤ 2.000	Profissional do sexo
	Laís (54)	SI	SI	Branca	EM completo	SI	Locutora
	Alice (26)	Travesti	Heterossexual	Preta	EF completo	> 1.000 e ≤ 2.000	Profissional do sexo
	Giovanna (19)	SI	SI	Parda	EM completo	SI	Estagiária
	Larissa (18)	SI	SI	Parda	EM completo	SI	Autônoma na área de vendas
	Lorena (29)	Mulher trans	Heterossexual	Parda	EM completo	> 500 e ≤ 1.000	Auxiliar de produção
	Bianca (42)	Travesti	Heterossexual	Parda	EF incompleto	≤ 500	Cabeleireira
	Sabrina (18)	Travesti	Heterossexual	Preta	EM completo	≤ 500	Estudante
	Juliana (45)	SI	SI	Parda	EF incompleto	SI	Ajudante de cozinha
	Gisele (33)	SI	SI	Parda	EF incompleto	SI	Profissional do sexo
	Camila (43)	SI	SI	Parda	EF incompleto	SI	Profissional do sexo
	Daniela (42)	Travesti	Heterossexual	Branca	EF incompleto	> 500 e ≤ 1.000	Profissional do sexo/cozinheira
	Thais (20)	Travesti	Heterossexual	Branca	EM completo	> 500 e ≤ 1.000	Estudante
	Stefani (26)	Mulher trans	Heterossexual	Amarela	EF incompleto	> 1.000 e ≤ 2.000	Estudante
	Emanuelly (31)	Travesti	Heterossexual	Parda	EM completo	> 1.000 e ≤ 2.000	Profissional do sexo
Campo Grande	Isadora (26)	Mulher	Outra	Preta	ES incompleto	> 2.001	Assessora Parlamentar
	Anita (25)	Travesti	Homossexual/gay/lésbica	Parda	EM completo	> 1.000 e ≤ 2.000	Autônoma
	Isabelly (34)	Andrógina/GA/NB	Homossexual/gay/lésbica	Parda	ES incompleto	> 1.000 e ≤ 2.000	SI
	Lohanna (29)	Mulher trans	Heterossexual	Branca	ES incompleto	> 1.000 e ≤ 2.000	Vendedora
	Eduarda (29)	Andrógina/GA/NB	Pansexual	Branca	ES incompleto	> 2.001	Fotógrafa
	Lara (29)	Mulher trans	Heterossexual	Branca	EM completo	> 2.001	Diarista
	Manuela (20)	Travesti	Heterossexual	Parda	EM completo	> 1.000 e ≤ 2.000	Agente de *call center*
	Gabriela (37)	Mulher trans	Heterossexual	Branca	ES incompleto	> 2.001	Profissional do sexo
	Lia (27)	Travesti	Heterossexual	Parda	EM completo	> 1.000 e ≤ 2.000	Profissional do sexo
	Lívia (41)	Mulher trans	Heterossexual	Preta	ES incompleto	> 1.000 e ≤ 2.000	Professora
	Yasmin (36)	Mulher trans	Heterossexual	Branca	EM incompleto	> 1.000 e ≤ 2.000	Desempregada
São Paulo	Rafaelly (20)	Mulher trans	Heterossexual	Parda	EM completo	> 1.000 e ≤ 2.000	Atendente de restaurante
	Cláudia (40)	Travesti	Bissexual	Parda	EM completo	SI	Profissional do sexo
	Diana (37)	Travesti	Heterossexual	Parda	EM incompleto	> 1.000 e ≤ 2.000	Profissional do sexo
	Eva (39)	Travesti	Heterossexual	Preta	EM completo	> 2.001	Orientadora socioeducacional
	Fátima (37)	Mulher trans	Heterossexual	Parda	EF incompleto	> 1.000 e ≤ 2.000	Agente de limpeza urbana
	Janaína (29)	Travesti	Heterossexual	Parda	ES incompleto	> 2.001	Estudante/profissional do sexo
	Grace (48)	Travesti	Heterossexual	Preta	EM completo	SI	Recepcionista
	Guiomar (52)	Travesti	Heterossexual	Parda	EF incompleto	> 500 e ≤ 1.000	Profissional do sexo
	Helena (42)	Travesti	Heterossexual	Parda	ES incompleto	> 2.001	Técnica de enfermagem
	Iara (50)	Mulher trans	Heterossexual	Branca	EF incompleto	> 1.000 e ≤ 2.000	Desempregada/profissional do sexo
	Ingrid (19)	Mulher trans	Heterossexual	Parda	EM incompleto	> 500 e ≤ 1.000	Desempregada
	Júlia (31)	Travesti	Heterossexual	Preta	EF incompleto	> 1.000 e ≤ 2.000	Profissional do sexo

* Anos completos no momento da entrevista; ^†^Raça/cor de pele autorreferida. SI: Sem informação; ES: Ensino superior; EM: Ensino médio; EF: Ensino fundamental; MTF: *male to* female; GA: Gênero ambíguo; NB: Não binária.

As narrativas das MTT entrevistadas demarcam uma expressiva polarização de suas experiências de acesso e de uso de serviços de saúde e seu uso. Por um lado, explicitam cenas de discriminação, estigmatização e patologização de suas identidades e modos de viver, evidenciando, assim, um processo histórico de transfobia institucional reproduzido nas posturas e ações dos/as profissionais de saúde, bem como na organização dos serviços. Por outro lado, apresentam experiências satisfatórias, caracterizadas pelo respeito e por atendimentos adequados. Nesses relatos, as participantes identificaram uma mudança em curso, expressa na ampliação da disponibilidade de serviços ofertados e na melhoria do atendimento a MTT.

### Dificuldades no acesso e no uso de serviços de saúde

A transfobia é identificada em várias nuances da dinâmica social desenvolvida nos serviços, desde olhares direcionados a elas até ações explicitamente discriminatórias ou mesmo entraves na assistência. A discriminação é descrita como uma realidade já conhecida por entrevistadas de diferentes faixas etárias, raça/cor, escolaridade e atuação profissional. Elas percebem reações de estranhamento à sua presença e formas sutis de discriminação por parte de profissionais da saúde ou de demais usuários/as dos serviços. Além de constranger MTT, essas situações se tornam obstáculos à sua permanência nos serviços e afetam sua saúde, já que comprometem a efetividade do cuidado (Excertos 1-4, Quadro 2).

**Quadro 2:** Excertos selecionados das narrativas das participantes

	Participante (cidade)	Excerto da narrativa
1	Thais (Manaus)	[Já me senti discriminada em serviço de saúde] várias vezes. É constante, eu estou acostumada. A gente vai viver por muito tempo assim e não tem como mudar. (...) Sempre tem uma pessoa que te olha, vira o olho, te olha de um jeito estranho. Tu estás tão legal, estás num dia tão lindo, a pessoa te olha assim e acaba o teu dia só pelo fato de você ser quem você é. E dói.
2	Rafaela (Porto Alegre)	Eu tive uma consulta com um dermato e daí também me senti um pouco estranha. Ele era meio seco, tipo “mostra isso aqui, mostra ali”. E daí eu me senti um pouco constrangida com o meu corpo.
3	Isabella (Porto Alegre)	Tem muita gente que olha diferente para trans, sempre. Vamos supor, você está no posto de saúde, passam 10 pessoas na sua frente, você vai ficando por último. Eu já escutei isso, o que não pode acontecer, tem que ser igualdade para todos.
4	Guiomar (São Paulo)	Para a gente, travesti, é muito complicado porque você não sabe como vai ser naquele posto que te atende. Tem um segurança (...), você não vai saber como ele vai te receber porque você é travesti, se vai soltar uma piadinha no meio, se vai ser educado. Você não vai saber como você vai passar pela moça que vai te atender. Então você vai com a fé e com a coragem. Você já está doente, mas tem sempre um engraçadinho. (...) Eles já sabem que vão atender uma trans, já vem alguma coisa dizendo meu nome, meu nome de batismo e mostra o nome social embaixo. [O médico] falou alto [meu nome de registro] e tinha umas pessoas lá, eu ouvi algumas risadinhas. (...) Eu quero voltar, mas quem disse que eu quero voltar a passar com ele? Não quero. E olha que eu estou adiando [a volta] já tem 1 ano.
5	Eva (São Paulo)	Eu já passei por uma situação de olharem e me chamarem pelo nome do meu pai. E mesmo com o meu nome sendo identificado. (...) Eu esperei ela chamar outros nomes, aí eu me levantei, pedi na maior educação, eu pedi licença e falei assim: “olha, o nome que você está repetindo há algum tempo é o nome do meu pai. O meu nome é Eva. Você já virou o meu RG duas vezes e você já observou que eu sou uma travesti. Eu vou voltar, eu vou sentar e você vai pronunciar o meu nome”.
6	Carolina (Porto Alegre)	Ela teve uma certa restrição dentro do hospital, onde, na época, a Lucy teve que buscar o Ministério Público, aquela coisa toda, onde deixaram ela numa área masculina mesmo ela sendo (...) redesignada, entendeu?
7	Janaína (São Paulo)	Tem uns CTA por aí, que eu conheço, que as meninas vão lá, e o médico, por exemplo, (...) não especifica como que vai funcionar a PrEP. Se ela sentir algum enjoo, quando der muito enjoo por conta que tomou hormônio, se pode tomar Plasil, se pode tomar algum remédio (...). E a gente sabe que o grau de escolaridade da população de travestis e transexuais não é alto. (...) Eu entendo, eu pergunto, mas tem meninas que têm até vergonha de perguntar. (...) Tem muitas meninas que eu conheço, principalmente na Zona Norte, que são de menor e que morrem em decorrência do HIV, da aids, na verdade. Então, eu acho que poderia melhorar essa questão também de fazer alguma coisa que fosse para as meninas que são de menor, que são profissionais do sexo, que vivem disso, (...) que são moradoras de rua (...) e não têm nenhum documento. É negado [a elas] também fazer isso [tratamento].
8	Laís (Manaus)	Eu sempre sou muito bem atendida quando eu vou ao SPA [Serviço de Pronto Atendimento] de São Raimundo. (...) Eu procuro lá porque é uma unidade que, toda vez que eu chego, eu nunca fui discriminada e nunca fui deixada de ser atendida. Sempre me recebem muito bem.
9	Eva (São Paulo)	Os serviços especializados são muito mais humanizados do que outros serviços, do que uma UBS [Unidade Básica de Saúde], do que um pronto-socorro, uma outra unidade de saúde. Porque os serviços especializados estão já mais acostumados com essa população em específico, que eu acho que é a população que mais se testa. E eles têm um treinamento para lidar com essas pessoas.
10	Manuela (Campo Grande)	Eu acho que deveriam acontecer mais coisas iguais ao que aconteceu comigo, como simplesmente aparecer alguém e dizer “está tendo exame lá, você tem que aparecer lá tal dia, tal hora” e ainda vai ter o incentivo [ajuda de custo], porque eu acho que não se é falado tanto sobre isso entre essa comunidade. Eu acho que falta um pouco de incentivo para que as meninas queiram fazer o exame.
11	Valentina (Manaus)	Eu gostei daqui. Inclusive, quando eu vim mês passado aqui no projeto (...), eu fui vacinada aqui no mesmo dia. Eu achei uma coisa assim tão rápida, eu passei pelos médicos, fiz os meus exames, os meus testes rápidos aqui. Eu achei muito positivo, interessante, vocês estarem junto ao ambulatório transgênero. Eu gostei assim, é um incentivo de trazer outras pessoas para cá, outras meninas que estão por aí, fora do sistema, não estão se tratando, não estão tendo acesso, para elas saberem também.
12	Cláudia (São Paulo)	Antigamente era mais preconceito, a gente sofria mais. Hoje em dia não, o negócio hoje está dez para a gente. A gente está tendo mais oportunidade de viver.
13	Júlia (São Paulo)	Os profissionais estão mais atentos porque hoje em dia [desrespeito] não passa batido, o gerente já chama na sala e fala: “então, gata, não vai rolar porque assim não dá; (...) você pode ser crente lá na igreja, aqui é UBS, aqui é o seu trabalho; então, se controle, respeita as pessoas”. É uma questão de respeito.
14	Laura (Porto Alegre)	Olha, eu acho que agora, hoje em dia, nós estamos tendo muito mais oportunidades, entendeu? Por exemplo, assim, agora abriu laboratório. (...) Lá eles fazem tudo que é tipo de tratamento para trans, travestis, sabe? E eles são bem atenciosos. Eu adorei. Eu fui lá. Eles são maravilhosos com a gente, sabe?
15	Heloísa (Porto Alegre)	O que falta é (...) atenção, agilidade nos exames para nós sabermos se estamos com HIV, se estamos com aids, se estamos com sífilis, com HPV, com gonorreia, com todas essas doenças. (...) Então, o que falta é uma hormonização e um projeto que possa assegurar para nós, trans e transexuais, ser mais tratadas, ser mais cuidadas, ser mais chamadas com rapidez.
16	Grace (São Paulo)	Muitas meninas trabalham na rua e estão muito expostas. Não dá, às vezes, para esperar 3, 4 meses. Está ali se expondo todos os dias e acontece, sei lá, de contrair alguma coisa e precisa de um atendimento mais rápido. Talvez fosse isso, a melhora ser isso, ter mais locais especializados para mulheres transexuais e travestis e homens trans também.
17	Eva (São Paulo)	Então, assim, é humanizar o atendimento. (...) Se a minha equipe não entende o que é a travestilidade e a transexualidade, eu, como direção desse espaço, eu ia buscar alguém que tenha maior e melhor conhecimento do que a minha equipe, que venha para treinar a minha equipe. [...] É tirar aquela coisa robótica, aquela coisa automática.
18	Rafaelly (São Paulo)	[Deveria ter] mais tratamento para pessoas trans. Mais alternativas. Ter em todos os postos endócrinas especializadas em pessoas trans, que eu acho que é isso que falta, elas saberem mesmo o que é um corpo trans e não achar que qualquer remédio, qualquer coisa vai nos ajudar.
19	Lia (Campo Grande)	Seria começando por uma capacitação dos profissionais para receber essas pessoas. [É] por causa, muitas vezes, de exclusão social que essas pessoas acabam não procurando o serviço de saúde e eu acho que, investindo em algumas alas específicas para a saúde de pessoas trans... Que existe uma especificidade: questão de hormonização, psicológico, enfim.
20	Fernanda (Porto Alegre)	Se as transexuais fizessem [os atendimentos] com elas próprias, delas para elas, elas não se sentiriam um extraterrestre dentro do hospital. Se a atendente fosse uma transgênero, se o médico fosse um transexual, elas iam ter um pouco mais de liberdade e se sentiriam pessoas normais. Eu acho que, se falassem a mesma língua... (...) Eu acho que, se abrissem portas para transexuais e transgêneros serem atendentes, auxiliar de enfermagem ou até mesmo médico, a comunicação ia ser um pouco mais clara.
21	Júlia (São Paulo)	A sociedade tem uma dívida. [O atendimento à população trans] é preferencial, sim, na saúde. Tem essa retratação histórica de que o SUS já matou muita gente. Então vai ter que tratar a gente como rainha, sim, na UBS, para tentar essa retratação, vai ter que chamar de dona, senhora, de madame, se for o caso. E vai ter que fazer um atendimento de qualidade. Por quê? Porque travesti é gente como todo mundo, só que o único diferencial que a gente tem é que a gente tem que viver provando que a gente é gente.

São recorrentes os relatos de que profissionais da saúde e demais trabalhadores/as dos serviços persistem no uso do nome de registro, mesmo quando cientes de como as MTT gostariam de ser chamadas. As entrevistadas percebem que os/as profissionais têm dificuldade ou resistência à efetivação do ajuste dos nomes, pronomes e referências de gênero em documentos, sistemas e na comunicação estabelecida nos serviços. Em alguns casos, o desrespeito ao nome social/retificado e aos pronomes de sua preferência revela, além de obstáculos no reconhecimento das identidades trans, uma intenção de inibir ou punir a transexualidade-travestilidade (Excerto 5, Quadro 2).

Outras dificuldades podem ser impostas a partir de ações que desconsideram as especificidades das MTT atendidas nos serviços. As MTT relataram se sentir desrespeitadas e desassistidas ao receberem atendimentos formatados para pessoas cisgênero ou enquadrados ao sexo registrado ao nascimento. Inclusive, em alguns relatos é possível identificar indícios de abuso de poder e tentativas de subjugação por meio da imposição de determinadas condições de atendimento. Na mesma direção, tornam-se limitados ou mesmo ineficazes os atendimentos que desconsideram as condições sociais precárias que afetam a saúde e as possibilidades de cuidado de algumas MTT (Excertos 6 e 7, Quadro 2).

### Ampliação de serviços e melhoria no atendimento

O relato de boas experiências em serviços de saúde aponta para iniciativas que buscam contrabalancear as iniquidades sociais e de saúde que atingem as MTT. A esse respeito, as entrevistadas destacam, como fatores decisivos para um bom atendimento, o respeito à identidade de gênero e a preparação da equipe profissional no cuidado dessa população. Tais critérios se mostram mais relevantes do que outras características, como a localização geográfica — o que faz, por exemplo, algumas MTT se deslocarem para bairros distantes de suas residências para receberem um atendimento adequado (Excertos 8 e 9, Quadro 2). Apesar da menção a serviços como Pronto Atendimento e Unidade Básica de Saúde, há um destacado reconhecimento do trabalho desenvolvido nos serviços especializados em ISTs/HIV/aids ou em transição de gênero, no que diz respeito aos critérios mencionados. Esse destaque não foi atribuído a categorias profissionais específicas.

A referência às experiências vividas nos serviços parceiros do projeto do qual deriva este estudo aponta para a relevância de estratégias utilizadas com propósito de:

a) ampliar a divulgação dos serviços nas redes de MTT;
b) viabilizar a ida aos serviços, especialmente daquelas para quem o custo com deslocamento pode ser impeditivo da busca por cuidado;
c) otimizar a presença da usuária no serviço, expandindo as tecnologias ofertadas e mitigando a demora e a fragmentação das intervenções — problemas típicos do sistema de saúde, que, no caso de populações menos vinculadas aos serviços, podem ser decisivos para a não continuidade do cuidado (Excertos 10 e 11, Quadro 2).

As boas experiências marcam um contraponto às dificuldades que caracterizam a relação das pessoas trans com a assistência em saúde ao longo do tempo. Especialmente as entrevistadas mais velhas percebem avanços importantes quando se referem ao crescente reconhecimento do direito dessa população à saúde; à ampliação da produção de conhecimento e do debate a respeito de suas questões de saúde; à maior disponibilidade de serviços direcionados a elas e/ou atentos a suas especificidades; e à melhoria na capacitação de profissionais para o atendimento a essa população (Excertos 12-14, Quadro 2).

A expectativa das entrevistadas é de que haja uma tendência de ampliação dos serviços, dos horários de atendimento e da disponibilidade de tecnologias, assim como de melhoria no atendimento a pessoas trans (Excertos 15 e 16, Quadro 2). A ideia de capacitação ou qualificação dos/as profissionais de saúde está presente nas narrativas como parte fundamental da mudança em curso. Ela é apresentada tanto no sentido de promover posturas e ações mais respeitosas quanto no de melhorar o entendimento acerca da transexualidade-travestilidade e o conhecimento técnico necessário para atender as necessidades de saúde dessa população (Excertos 17-19, Quadro 2).

A mudança vislumbrada no acesso e uso dos serviços de saúde é endereçada, por fim, às questões estruturais que historicamente desencadeiam iniquidades sociais e em saúde, refletidas, por exemplo, na escassez de pessoas trans nas esferas de elaboração e condução das políticas e ações de saúde, de modo que o cuidado em saúde fica quase exclusivamente a cargo de pessoas cisgênero, em geral, referenciadas pela própria cisgeneridade (Excertos 20 e 21, Quadro 2).

## DISCUSSÃO

A análise das dificuldades enfrentadas pelas MTT entrevistadas quanto ao acesso e ao uso dos serviços e ao seu uso reafirma o panorama presente na literatura, que mostra a abrangência das iniquidades de saúde, que derivam da transfobia estrutural, bem como seu impacto na saúde e na vida de MTT^1,3,13,21-23^. Nesse sentido, é evidente uma contradição ético-prática, presente nos espaços de cuidado à saúde. Quando buscados por MTT, na expectativa de alívio ou prevenção de adoecimento, esses espaços, muitas vezes, se apresentam como ambientes hostis, que as repelem em vez de acolhê-las, aumentando sua vulnerabilidade a diferentes agravos.

Muitas dessas dificuldades são relacionadas especificamente à discriminação sexual e de gênero, embora as MTT também enfrentem obstáculos comuns a outros/as usuários/as do SUS, como filas, falhas na informação e ausência de médicos^18^. A recorrência do desrespeito ao nome social/retificado e à identidade de gênero não apenas incorre na negação de direitos conquistados^2^, mas se torna um obstáculo na busca de serviços e causa abandonos de tratamentos^13,14,21,24^.

Os indícios de punição e subjugação, identificados nas posturas de profissionais de saúde descritas pelas entrevistadas, mostram como conflitos acerca das concepções de gênero e disputas no campo da moral sexual compõem a dinâmica social dos serviços de saúde. Assim, a presença de MTT nos serviços implica possíveis tensões e confrontos, o que as afasta dos serviços de saúde. A transfobia também pode ser identificada de forma sistêmica na escassez de formação e de competência dos/as profissionais de saúde para lidarem com as identidades, questões e necessidades de saúde específicas das pessoas trans^16,23^. Consequentemente, é alimentado um círculo vicioso no qual se articulam as expressões sociais da transfobia, a escassez de políticas públicas que visem garantir os direitos de MTT e a pouca preparação de serviços e profissionais de saúde para atendê-las. Embora as dificuldades no acesso e no uso dos serviços possam se articular a outras dinâmicas sociais discriminatórias, como o racismo^25^, as entrevistas não se ativeram a essa relação.

As boas experiências, por sua vez, revelam avanços na construção de uma assistência à saúde que respeite as expressões e especificidades das MTT e atenda de forma adequada às suas necessidades de cuidado. A percepção de que as possibilidades e condições de acesso e uso dos serviços de saúde estão mudando ainda é pouco referida na literatura e reflete o impacto da recente ampliação da disponibilidade de serviços oferecidos a MTT e da melhoria do atendimento na saúde e na vida dessa população.

Por um lado, a expectativa de que tais ampliações e melhorias sejam continuadas aponta que os avanços identificados ainda são insuficientes para mudar o cenário da histórica exclusão vivida por MTT nos serviços de saúde. Por outro, essa expectativa reafirma alguns caminhos que se mostram promissores. A referência ao bom atendimento recebido, especialmente nos serviços especializados em ISTs/HIV/aids, de transição de gênero e nos serviços parceiros desse projeto, endossa a relevância das estratégias adotadas nesses espaços com o intuito de combater a discriminação e oferecer ações direcionadas às especificidades de saúde das MTT e ao alívio das iniquidades sociais que as afetam.

A mudança identificada nessa direção tem sido empreendida na interface das políticas públicas de saúde com o ativismo LGBT+ e a produção de conhecimento acerca das questões de saúde dessa população. Resulta, portanto, do aumento de reconhecimento e visibilidade social das MTT no Brasil a partir de sua afirmação política^18^, assim como da recente expansão do debate acadêmico relativo a esse grupo. Segundo Favero^26^, os “estudos trans”, que se propagaram no país nos anos 2000, ultrapassaram o foco na prostituição de MTT e abriram a discussão sobre saúde e clínica, especialmente nas disputas pela despatologização do gênero no âmbito dos guias diagnósticos. Com o transfeminismo^3^, houve uma atualização das formas de organização política dessa população para além da atuação em ONGs ou associações ligadas ao enfrentamento da epidemia de HIV/aids. Nesse sentido, a maior presença de MTT nas universidades produziu um importante deslocamento da posição de pesquisadas para a de pesquisadoras, junto com a reivindicação de reconhecimento epistemológico de suas cosmologias, incluindo suas noções de adoecimento e bem-estar. Em consonância, algumas entrevistadas enfatizam que a superação das dificuldades de MTT nos serviços de saúde está atrelada à necessidade de romper as barreiras que as excluem das esferas de elaboração e condução das políticas e ações de saúde, quase sempre referenciadas pela cisgeneridade.

Como o desenho deste estudo implicou o uso dos serviços pelas participantes, o relato das dificuldades enfrentadas pode ter sido atenuado frente à transposição de algumas possíveis barreiras de acesso. Além disso, apesar das estratégias de divulgação e de convite para participação, podem não ter sido incluídas MTT em situações mais extremas de vulnerabilidade, especialmente aquelas poucos inseridas em redes de sociabilidade ou residentes em áreas com menos infraestrutura (com carência de transporte público, internet etc.). Comparações entre os resultados por região não foram possíveis devido a especificidades na realização da pesquisa em cada sítio, especialmente no que diz respeito às adaptações necessárias ao enfrentamento da pandemia de COVID-19.

As narrativas analisadas mostram o entrecruzamento de dois movimentos opostos e conflitantes na relação das MTT com os serviços de saúde. Essa oposição ganha contornos mais acentuados diante da atual conjuntura política e social brasileira. O recrudescimento do conservadorismo nos últimos anos — com a escalada do fundamentalismo religioso no Estado e as ofensivas contra os estudos e o debate de gênero e sexualidade — resulta no aumento da transfobia em suas diferentes manifestações e no cerceamento da cidadania das pessoas trans^27^. Em contrapartida, a retomada democrática pós-pandemia, com a eleição de MTT para cargos políticos e a reestruturação das políticas públicas de saúde e de Direitos Humanos, abre espaço para que os avanços conquistados ou em perspectiva sejam efetivados ampla e consistentemente, de modo a mudar, de fato, as condições de saúde das pessoas trans.

## Footnotes

1 Os autores do *Manual de Cuidado* (*Standards of Care*), produzido pela *World Professional Association for Transgender Health’s* (WPATH), em sua oitava versão (2022), utilizam o termo *Transgender and Gender Diverse* (TGD) para abranger a maior variedade global possível de pessoas com identidades e expressões de gênero que diferem do gênero atribuído a elas no nascimento^1^. Neste artigo, é priorizado o uso do termo trans, que tem sido utilizado no Brasil como categoria englobante dessas diferentes identidades e expressões de gênero^2^.

2 A Portaria n^o^ 1.820, de 13 de agosto de 2009, do Ministério da Saúde, estabelece como direito, na rede de serviços de saúde, o atendimento humanizado, acolhedor, livre de qualquer discriminação, restrição ou negação em virtude de identidade de gênero, entre outras características. Resolve também que deve existir em todo documento da/o usuária/o um campo para se registrar o nome social independente do registro civil, sendo assegurado o uso do nome de preferência (Disponível em https://bvsms.saude.gov.br/bvs/saudelegis/gm/2009/prt1820_13_08_2009.html).

3 O transfeminismo consiste em uma linha de pensamento e de prática feminista fundamentada no processo de consciência política e resistência das pessoas trans, assim como no feminismo negro, principalmente nos conceitos de interseccionalidade e não hierarquia de opressões. Rediscute a subordinação morfológica do gênero ao sexo, que tem servido como justificativa para a opressão de pessoas cujos corpos não estão em conformidade com a norma binária homem/pênis e mulher/vagina^28^.
